# Palliative Care Consultation and Takeover Models: Cut Your Coat According to Your Cloth

**DOI:** 10.1089/pmr.2024.0025

**Published:** 2024-05-15

**Authors:** Mohammad Zafir Al-Shahri

**Affiliations:** Department Palliative Medicine, Alfaisal University, Riyadh, Saudi Arabia.

*Dear Editor:*

I read with great interest the article by Dr. Pereira and colleagues describing the palliative care (PC) consultation–shared care–takeover (CST) framework.^1^ My interest in this article did not solely stem from the fact that it approaches a common challenge in the field but also from our local experience of trying to mitigate it. In my brief commentary on this thought-provoking article, I will share some views on the CST framework and then refer to our local case.

The authors have described the development of the CST framework to facilitate the understanding and monitoring of the model of care adopted by the specialist PC teams when they collaborate with referrers. The CST framework represents three models: first is *consultation*, where the PC team provides PC advice and support to the primary care team; second is the *shared care*, where the PC team assumes the primary care role for PC needs while the referrer team remains the primary care provider for other needs; third is the *takeover*, where the PC team becomes the primary care provider for all needs. The most responsible practitioner (MRP) is the attending practitioner representing the primary care team.

The *shared care* model remains part of the framework, despite the authors’ recognition of its rarity and impracticality. The framework presented it as a distinct model when, in reality, it merely represents a more intensified *consultation* model. In practical terms, having more than one MRP for a given patient at a given time is inconceivable. That is probably why the authors referred in the *shared care* model to a PC clinician and an MRP instead of two MRPs. Needless to say, how problematic it would be when two MRPs caring for the same patient have conflicts of interest for various reasons such as remuneration system issues or resource–service imbalances, let alone differences in the specialty and schools of thought. But let us assume that a PC patient cared for at home by his family has two MRPs who were family physicians (one with PC and one without PC-added competency certification) with a perfect collegial relationship. How feasible would it be to clarify to the family caregivers which MRP to call for every possible problem they may encounter during caregiving? Indeed, the authors rightly referred to some concerns that having more than one MRP may create confusion and risk patients’ safety. Given the above, the CST framework may be better off without the *shared care* model.

Also, subcategorizing the *consultation* and the *takeover* models does not seem necessary. Without delving into details, most of the subcategories suggested in the framework are either identical or characterized by minimal differences that do not justify subcategorization. For example, in the *consultation* model, three follow-up visits represent a different subcategory from several visits. That seems to lead to nothing else but unnecessary inflation of the framework.

More importantly, the authors listed some real-life cases to prove the utility of the CST framework in practice. Upon reviewing the listed cases, none was convincing of the utility of the CST. PC practitioners do not need to review medical records, use the CST framework, and plot data on radar graphs to understand what model of care they follow—*consultation*, *takeover*, or a combination. If anything, the illustrative cases stated the obvious by implicitly concluding that staffing adequacy determines whether a PC program should be limited to the *consultation* model or include the *takeover* model.

In this context, I would like to share our experience in a tertiary hospital where our PC program provides inpatient, outpatient, and home care services using *consultation* and *takeover* models. At some stage, the increasing demand for the *takeover* model by the referring teams motivated us to develop a referral policy that took a couple of years of discussions with the stakeholders before becoming an approved hospital policy. The policy is widely open for referrals to PC at the *consultation* level but requires meeting specific criteria at the *takeover* level. After several years of implementing the PC referral policy, we investigated its impact.^2^ The results suggested that the referral policy helped referrers to make timely and better-organized *takeover* referrals but failed to encourage them to make early *consultation* referrals. After many years of implementing the PC referral policy, we had a period of staffing crisis because of the resignation of several expatriate physicians. We responded to the challenge by keeping the *takeover* model temporarily on hold until the hospital resolved the staffing crisis.

The message I intend to share based on the above discussion is that PC practitioners have much less difficulty knowing what model of care they are following than knowing what would be the optimal referral criteria that ensure delivering the best possible PC service following the most suitable model of care, given the resources they have.

## Abbreviations Used

CST: consultation-shared care- takeover
MRP: most responsible practitioner
PC: palliative care
